# The Role of a Spiritual Approach in Patients With Cancer: A Systematic Review

**DOI:** 10.7759/cureus.89783

**Published:** 2025-08-11

**Authors:** Carolina Jorge Gonçalves, Ana Sofia Almeida

**Affiliations:** 1 Family Medicine, Unidade Local de Saúde de Entre Douro e Vouga, Santa Maria da Feira, PRT

**Keywords:** cancer, quality of life, spirituality, symptoms, well-being

## Abstract

Health encompasses physical, psychosocial, cultural, economic, and spiritual well-being. Spirituality is a dynamic, personal dimension that, when nurtured, can enable the individual to feel balanced (with themselves, others, and the environment). The increase in chronic diseases and, in parallel, the growing need for palliative care (PC) are undeniable. Given the scarcity of such care, it is important that primary healthcare (PHC) addresses this type of need, with a particular focus on spirituality.

The aim of this study is to assess the role of a spiritual approach in terms of symptom control, disease adaptation, or quality of life in patients with cancer.

A systematic search was conducted in PubMed (MEDLINE), Cochrane Library, EMBASE, and PsycINFO. MeSH terms (or keywords when not applicable) were used: "Neoplasms" AND “Spiritual Therapies” AND ("Stress, Psychological" OR "Pain" OR "Quality of Life"). A total of 722 articles were obtained, and after a phased analysis, involving the review of titles, abstracts, and full-text documents, seven studies were selected. Risk of bias was assessed using the “Cochrane RoB 2.0” tool. The Strength of Recommendation Taxonomy (SORT) tool was applied to evaluate the quality of evidence. Preferred Reporting Items for Systematic Reviews and Meta-Analyses (PRISMA) guidelines were followed.

All analyzed articles were randomized controlled trials, reporting favorable outcomes for interventions (such as mindfulness programs, religious practice, the combination of both, and transcendental meditation). Overall, across the studies and despite the use of different scales, statistically significant differences were found in several outcome dimensions (including quality of life, symptoms - anxiety, depression, pain, nausea and vomiting - spiritual well-being, and suffering), all in favour of the various spiritual approaches.

In line with other synthesis studies, the results of this review support the benefits provided by a spiritual approach (in its various dimensions). The risk of bias in the analyzed studies was qualitatively classified as “low risk” in most domains and “some concerns” in only one domain, which affected the overall assessment. Noted limitations include the heterogeneity of the interventions compared, as well as the multiple scales used. According to the SORT tool, the studies in this review were determined to be Level 1 evidence and the conclusions were given a Strength of Recommendation A; therefore, they should be considered in clinical practice.

In summary, a beneficial effect of a spiritual approach in the complementary management of patients with cancer can be recognized. The findings support the need to expand the PC network and the relevance of its interdisciplinary nature. It also reflects on the importance of including spirituality in the practice of PHC, which is often the provider of longitudinal care until the end of life.

## Introduction and background

Health status is not merely the absence of disease; it includes various dimensions, namely physical, psychosocial, cultural, economic, and spiritual well-being [1]. In accordance with this, existential well-being is strongly associated with the concept of health in its broadest sense [2]. There are multiple definitions of spirituality, with a general consensus that it is a personal dimension, unique to each human being, that integrates their life experience and enables transcendence (towards oneself, others, or higher entities/forces). It grounds the individual in the search for meaning, goals, and life purpose, which are fundamental to continuously building a balanced state of mind and inner peace [3]. Religiosity may be a form of spirituality, yet these constructs are distinct, with the latter being much broader. Religious practice is associated with specific beliefs and rituals and has its own symbols [4].

With advancements in public health and medical science, chronic diseases have become the predominant cause of morbidity and mortality, making increased investment in the care provided to individuals affected by these conditions essential. In this context, it is important to mention the role of palliative care (PC), whose main objective is to promote the well-being and quality of life (QoL) of the patient and their family through the prevention and relief of physical, psychological, social, and spiritual suffering caused by incurable or severe illness in advanced and progressive stages, using active, coordinated, and holistic care approaches [5].

Given the vast population that should have access to PC, and the evident shortage of dedicated teams (even for oncological conditions), and according to what the World Health Organization (WHO) states, i.e., primary healthcare (PHC) can meet most of a person’s health needs throughout life (including prevention, treatment, rehabilitation, and palliative care) [6], PHC professionals should be alert to this area, with spirituality being one of the domains that should be addressed.

This topic has been investigated in several original studies, making this systematic review relevant. Its objective is to evaluate the role of the spiritual approach in patients with cancer, in terms of symptom control, disease adaptation, or QoL. Thus, this systematic review distinguishes itself from those previously published in the literature by not restricting the type of spiritual intervention or approach.

## Review

Methods

Research Strategy

As a systematic review, the following PICO was defined: Population - patients with cancer; Intervention - spiritual approach; Comparison - usual care; Outcome - symptom control, disease adaptation, or QoL. In April 2024, a search was conducted in the following databases: PubMed [MEDLINE], Cochrane Library, EMBASE, and PsycINFO. The research question was constructed using the following MeSH terms (or keywords when MeSH terms were not applicable): "Neoplasms" AND “Spiritual Therapies” AND ("Stress, Psychological" OR "Pain" OR "Quality of Life").

Study Selection

The researchers included studies that examined the impact of spiritual approaches, such as mindfulness, transcendental meditation, religious practices, or structured spiritual therapies, on symptom control, disease adaptation, or QoL in patients with cancer. Only randomized controlled trials (RCTs) with a control group and using validated assessment instruments were considered. No temporal or language filters were applied. Studies were excluded if they were non-randomized, qualitative, case reports, and reviews, involved non-oncological participants or individuals under the age of 18, or lacked a clear description of the spiritual intervention or outcomes of interest.

Title and abstract screening was conducted independently by two researchers. Full texts of potentially eligible articles were then reviewed. Data extraction was performed individually and independently using a standardized form. Extracted data included author and year of publication, country, study design, type and duration of the intervention, number and characteristics of participants, assessment instruments used, and main findings. Any disagreements, which amounted to approximately 5%, were resolved through discussion and consensus among the researchers.

**Figure 1:**
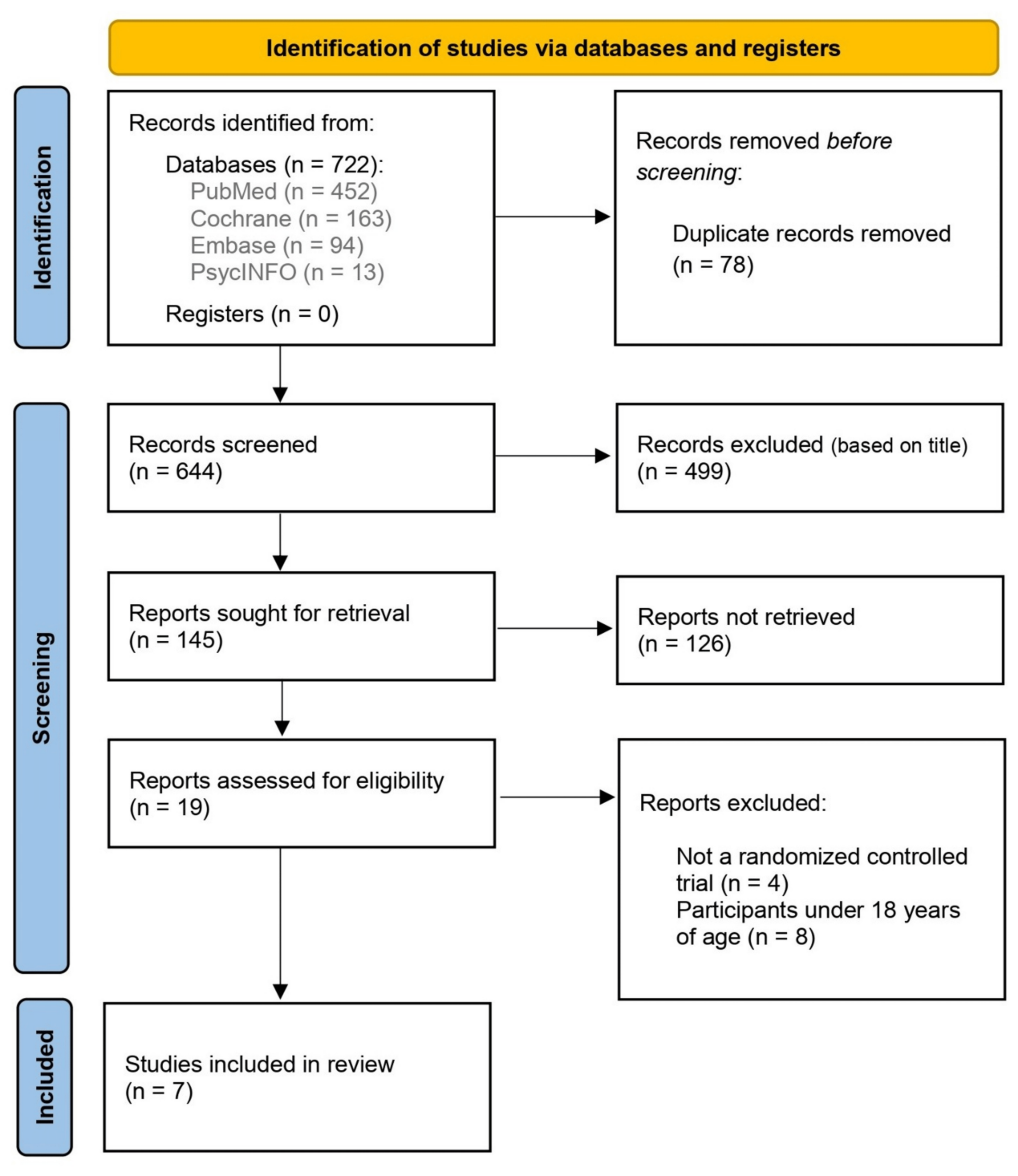
The PRISMA Figure Showing the Steps to Choose the Studies for Systematic Review PRISMA: Preferred Reporting Items for Systematic Reviews and Meta-Analyses.

Across the databases, 722 articles were retrieved. Duplicates were removed and, following a review of all titles, 145 were selected. From these, 19 studies were selected for full-text reading based on abstract analysis. In the end, seven articles of interest were included (Figure 1).

Data Extraction

To conduct a structured analysis of the studies, the authors created a table with the main aspects of each (Table 1). Given the sample composition, for all RCTs, the “Cochrane RoB 2.0” risk of bias assessment tool was used (Table 2). Additionally, the Strength of Recommendation Taxonomy (SORT) was applied to determine the level of evidence and strength of recommendation. The investigators independently collected and analyzed the studies and then discussed any disagreements. The manuscript was written in accordance with the Preferred Reporting Items for Systematic Reviews and Meta-Analyses (PRISMA) guidelines, along with the standards of the Revista Portuguesa de Medicina Geral e Familiar (RPMGF).

**Table 1:** Summary of Included Studies This table presents the main characteristics of the seven randomized controlled trials (RCTs) included in the systematic review. For each study, the location, design, sample size, type of spiritual intervention implemented, and main outcomes are summarized. Interventions include mindfulness-based programs, spiritual therapy, religious practice, and transcendental meditation. All studies reported statistically significant improvements in at least one outcome related to quality of life (QoL), symptom control (e.g., pain, anxiety, depression), or spiritual well-being in patients with cancer.

Reference	Location	Design	Sample Size	Intervention	Main Results
Nidich et al. (2009) [7]	USA	RCT	130	Transcendental meditation	Improved QoL and emotional and social well-being
Foley et al. (2010) [8]	Australia	RCT	115	Mindfulness-based cognitive therapy	Improved depression, anxiety, distress, and QoL
Jafari et al. (2013) [9]	Iran	RCT	68	Spiritual therapy sessions	Improved QoL and functional scales
Würtzen et al. (2013) [10]	Denmark	RCT	336	Mindfulness-based stress reduction	Reduced anxiety and depression
Würtzen et al. (2015) [11]	Denmark	RCT	336	Mindfulness-based stress reduction	Reduced distress, improved mindfulness, and spiritual well-being
Oner Cengiz et al. (2023) [12]	Turkey	RCT	65	Mindfulness-based therapy	Improved QoL and spiritual well-being
Sabet et al. (2023) [13]	Iran	RCT	80	Spirituality-based palliative care	Improved QoL, reduced pain, and nausea/vomiting

**Table 2:** Risk of Bias Assessment (Cochrane RoB 2.0) This table summarizes the risk of bias evaluation for each of the seven included randomized controlled trials (RCTs), following the Cochrane RoB 2.0 tool. It assesses five domains: bias arising from the randomisation process, deviations from intended interventions, missing outcome data, measurement of the outcome, and selection of the reported result. The overall judgment for each study is presented. Most studies showed low risk in all domains except for outcome measurement or selective reporting, where “some concerns” were identified due to limitations in blinding or self-reported data.

Study	Randomization	Intervention Deviations	Missing Data	Outcome Measurement	Selective Reporting	Overall Bias
Nidich et al. (2009) [7]	Low	Low	Low	Low	Low	Low
Foley et al. (2010) [8]	Low	Low	Low	Low	Some concerns	Some concerns
Jafari et al. (2013) [9]	Low	Low	Low	Low	Some concerns	Some concerns
Würtzen et al. (2013) [10]	Low	Low	Low	Low	Some concerns	Some concerns
Würtzen et al. (2015) [11]	Low	Low	Low	Low	Some concerns	Some concerns
Oner Cengiz et al. (2023) [12]	Low	Low	Low	Low	Some concerns	Some concerns
Sabet et al. (2023) [13]	Low	Low	Low	Low	Some concerns	Some concerns

Results

Of the seven studies analyzed, four involved mindfulness programs as the intervention, one focused on religious practice, another combined these approaches, and finally one involved transcendental meditation.

It is worth noting that mindfulness is characterized by the acceptance of internal and external experiences (based on Buddhism). In 1979, Jon Kabat-Zinn defined it as the ability to be present in the moment on purpose, without judgment. He developed a mindfulness-based stress reduction (MBSR) program, which consists of group intervention involving meditation practice, yoga, and education about the psychological and physiological aspects of stress. It typically consists of one weekly session lasting 2 to 2.5 hours, over a total of eight sessions, as well as a recommended period of silent practice at home [14].

In all the analyzed studies, it was reported that there were no significant differences in the demographic characteristics between groups, and an intention-to-treat analysis was consistently conducted.

Nidich et al. (2009) [7] conducted an RCT evaluating QoL in women aged ≥55 with stage II-IV breast cancer in Chicago. The intervention group (IG, n=64) underwent a transcendental meditation program (vs control group, CG, n=66), for up to two years, with a median follow-up of 18 months. QoL was assessed every six months using FACT-B, FACIT-SP, and SF-36 scales. Analysis was performed using repeated-measures ANCOVA to adjust for the unequal distribution of estrogen receptor status in the overall FACT-B scores. Results showed statistically significant improvements in overall QoL (total FACT-B score, p=0.037); emotional and social well-being (FACT-B subscales, p=0.046 and p=0.003, respectively); and mental health (SF-36 scale, p=0.017).

Foley et al. (2010) [8] reported an RCT in Australia involving patients with cancer aged ≥18 years, investigating the effect of participation in mindfulness-based cognitive therapy (MBCT). Of the 55 participants initially allocated to the IG, six participants were lost during the study, and six participants of the 60 in the CG were also lost. The intervention comprised eight MBCT sessions (one per week, lasting two hours), with a five-hour silent retreat between the sixth and seventh sessions. Five outcomes were assessed: depression, anxiety, distress, QoL, and mindfulness, at three time points (pre-intervention, post-intervention, and three months later). Notably, significantly greater improvements were seen in depression (p<0.001), anxiety (p=0.002), and distress (p<0.001) using HAM-D, HAM-A, and DASS scales, respectively. QoL, assessed using the FACT-G scale, improved significantly in the IG (p=0.011), and significantly higher mindfulness scores were observed in the IG using the FMI scale (p<0.001). Importantly, benefits were maintained in the IG at the three-month follow-up.

Jafari et al. (2013) [9] conducted an RCT in Iran including women with breast cancer diagnosed within the previous year and recommended for radiotherapy (RT), regardless of stage. Patients with concurrent chronic disease, major depressive disorder, or those missing two consecutive sessions were excluded. The CG had 31 participants vs 37 in the IG (with three dropouts). The intervention involved six weekly spiritual therapy sessions lasting 2-3 hours. Using a repeated-measures general linear model MANOVA, the IG showed significant improvements in global health status/QoL (Cohen’s d effect size: 2.16, 95% CI: 1.56-2.78), as well as statistically significant changes in all EORTC QLQ-C30 functional scales post-intervention (p<0.05). All symptoms improved in the IG on the QLQ-C30 scale, except for dyspnea, appetite loss, constipation, and diarrhea. On the EORTC QLQ-BR-23 functional scales, the IG showed improvement (except for sexual satisfaction), and the intervention reduced side effects of systemic therapy (p=0.02) and discomfort from hair loss (p=0.00) based on QLQ-BR-23 symptom scales.

Würtzen et al. (2013) [10] conducted an RCT in Denmark involving women aged 18-75 with stage I-III breast cancer, who had undergone oncological surgery 3-18 months prior. Exclusion criteria included active treatment for major psychiatric illness and diagnosis of other cancers in the past 10 years, among others. The IG included 168 participants (35 were lost to follow-up) vs 168 in the CG (18 lost to follow-up). The intervention consisted of eight MBSR sessions (one per week, two hours each) and a five-hour silent retreat after the seventh session. Anxiety and depression were assessed at four time points (pre-intervention, immediately post-intervention, and at six and 12 months).

A statistically significant reduction in depression was observed using the CES-D scale (p<0.05). At six months, a borderline difference was found for anxiety (p=0.05), and a statistically significant difference was found for depression, using both the SCL-90r (p=0.01) and CES-D (p=0.03) scales. At 12 months, the difference in depression remained statistically significant on the SCL-90r (p=0.03) but was borderline on the CES-D (p=0.05). When calculating the variation in scores (final vs baseline), a greater statistically significant change in depression was found in the IG using the SCL-90r (p=0.01), but this was not observed for depression using CES-D (p=0.22) or for anxiety (p=0.08).

Later, Würtzen et al. (2015) [11] conducted another RCT in Denmark with a similar study design: the same intervention strategy, but with different outcomes assessed (namely somatic symptoms, distress, core mindfulness skills, and spiritual well-being), requiring the use of different scales (BCPT, SCL-90r, FFMQ, and FACIT-Sp).

A reduction in somatic symptoms in the IG (vs an increase in the CG) was found to be statistically significant immediately post-intervention (p=0.01) and at six months (p=0.002), but not at 12 months (p=0.22). In terms of distress, a statistically significant reduction was observed in the IG at all time points post-intervention (immediately p=0.01, at six months p=0.001, and at 12 months p=0.04). A statistically significant improvement in mindfulness was found only at six months in the IG (p=0.01). Lastly, a statistically significant difference in spiritual well-being in the IG (vs CG) was observed at six months (p=0.02), but not at 12 months (p=0.11).

Oner Cengiz et al. (2023) [12] reported an RCT conducted in Turkey involving women aged 18-65 with breast cancer diagnosed at least six months prior. Exclusion criteria included stage IV cancer, need for inpatient treatment, the presence of another type of cancer or psychiatric illness. There were 32 participants in the IG and 33 in the CG. The intervention consisted of mindfulness-based therapy, with one 45-60 minute session per week over eight weeks, conducted via Zoom.

In this trial, QoL and spiritual well-being were assessed using the FACIT-Sp (Version 4) scale. Regarding QoL, no statistically significant difference was observed within either group when comparing baseline and final scores (p>0.05), although a positive variation occurred only in the IG. In the final comparison between the IG and CG, a statistically significant difference was found across multiple domains and in the overall score (IG: 66.98 ± 17.72 vs CG: 53.03 ± 18.65, p=0.00).

Concerning spiritual well-being, no statistically significant difference was found when comparing baseline and final scores within the IG (p>0.05), although again a positive trend was observed in the IG (vs a negative trend in the CG). In the final comparison between IG and CG, statistically significant differences were noted in the “Meaning” domain (p=0.01) and in the overall score (IG: 31.56 ± 8.90 vs CG: 27.09 ± 9.51, p=0.04).

Finally, Sabet et al. (2023) [13] conducted an RCT in Iran involving women aged 25-75 with stage I-III colorectal cancer who had received at least one cycle of chemotherapy (CT), had a functional status of at least light activity, and had no history of spiritual care through PC. Exclusion criteria included missing more than two sessions, lack of interest in participating, or incomplete questionnaires. The study population included 40 participants in both the IG and CG, with no dropouts.

The intervention involved four weekly two-hour sessions of spirituality-based PC. Three questionnaires were used to assess QoL, pain, and nausea/vomiting: WHOQOL-BREF, BPI, and PUQE, respectively. Statistically significant differences were observed between IG and CG for QoL (9.22 ± 0.61 vs 6.60 ± 1.58, p=0.001), pain (57.60 ± 7.06 vs 77.43 ± 5.50, p=0.001), and nausea/vomiting (4.80 ± 1.15 vs 8.20 ± 2.79, p=0.001).

Risk of Bias

To assess the risk of bias in the articles included in this review, the authors used the “Cochrane RoB 2.0” tool [15]. According to the data in Table 1 (characteristics and limitations of the trials, in which the reported losses to follow-up, for various reasons, did not appear to compromise randomization or internal validity), the biases ranged from “low risk” to “some concerns,” as shown in Table 2. It is worth noting that the “some concerns” judgement applied primarily to domain 4 (risk of bias in the measurement of outcomes), as it is possible that the outcome assessment was influenced by participants’ awareness of the intervention they received. Although there is no reason to believe this was likely, it cannot be ruled out, given that the participants were also the ones responding to the multiple questionnaires. As a result of the classification of “some concerns” in one domain, the overall risk of bias assessment was affected in six of the seven studies analyzed (except for that by Nidich et al. (2009) [7], in which blinding was applied).

Discussion

Multiple studies, including other systematic reviews and meta-analyses, support the benefits provided by spiritual approaches (in the various forms they may take). One such example is the systematic review by Smith et al. (2005) [16], regarding mindfulness-based stress reduction as a supportive therapy in cancer treatment, which analyzed three RCTs and seven uncontrolled clinical trials: benefits were noted at the psychological level (mood and stress), as well as in other areas associated with QoL, with no reported negative effects. More recently, Xing et al. (2018) [17] reinforced the value of this approach (through multiple practices) in a meta-analysis of 10 RCTs, involving 1,239 patients, which found improvements in spiritual well-being and QoL, as well as reductions in depression, anxiety, and hopelessness. Nevertheless, the author highlights the need for further studies, given the heterogeneity observed among the study designs analyzed.

Limitations

In this review, the authors highlight as limitations the unequal nature of the interventions across RCTs (although, given the broad scope of spirituality, such heterogeneity is acceptable and should be expected), and the disproportionate representation of approaches (predominance of mindfulness), as well as the evaluation of different outcomes using various and multiple scales. Additionally, it is worth noting that the original studies had sample sizes of differing magnitudes, which may not be sufficiently significant to ensure external validity. A further limitation may be the absence of a third reviewer to assist in resolving potential disagreements.

Implications

Using the SORT tool, the level of evidence of the articles included in this review was determined, with all classified as level 1 evidence, good-quality, patient-oriented evidence, according to several criteria. Thus, due to the consistency of the findings, the authors consider that the conclusions of this review carry a strength of recommendation A and, therefore, should be taken into account in clinical practice. Nonetheless, future research should be conducted with larger groups and standardized protocols, allowing for more reliable comparisons between results.

## Conclusions

With this review, evidence suggest that the spiritual approach has shown to play a beneficial and promising role in the complementary management of patients with cancer (in terms of symptom intensity, spiritual well-being, and QoL). This reinforces the need for greater investment in the creation of more interdisciplinary palliative care teams, as well as the importance of strengthening competence in the domain of spirituality and, for example, incorporating spiritual assessments into cancer care guidelines. PHC, being widely accessible, should always consider the relevance of the spiritual domain in the care provided to its users, in line with its holistic and comprehensive approach, as well as the longitudinal nature of its care.
